# Studies on Adsorption of Fluorescein Dye from Aqueous Solutions Using Wild Herbs

**DOI:** 10.1155/2020/8019274

**Published:** 2020-01-16

**Authors:** Ghadah M. Al-Senani, Nada S. Al-Kadhi

**Affiliations:** Department of Chemistry, College of Science, Princess Nourah Bint Abdulrahman University, Riyadh, Saudi Arabia

## Abstract

The adsorption of fluorescein dye (FD) on wild herb microparticles (*Juniperus* (JH) and *Solenostemma argel* (Del) Hayne (SH)) was studied to elucidate the changes in adsorption behavior with various parameters, such as initial concentration, adsorbent dosage, pH, contact time, and temperature. It was determined that the adsorption percentage of JH for FD was as high as 85.5%, which was higher than that of SH (71.9%). The morphologies of JH and SH were analyzed using Fourier-transform infrared spectroscopy (FTIR), X-ray diffraction (XRD), and scanning electron microscopy (SEM) analyses. The JH and SH adsorbents contained different functional groups, which were involved in the binding of the FD molecules during adsorption. The XRD patterns of JH and SH confirmed the presence of a combination of amorphous and crystalline phases in their structures. The SEM images of the surface of JH revealed the presence of deep pores ranging in size from 1.9 to 3.5 *μ*m, while SH contained smaller pores ranging in size from 130 to 350 *μ*m, which could help absorb large quantities of FD. The Freundlich model fitted the adsorption isotherms better than the Langmuir model. The values of the Freundlich equilibrium coefficient and separation factor ranged from 1 to 2 and from 0 to 1, respectively. The maximum adsorption capacities of JH and SH were determined to be 2.91 and 2.565 mg/g, respectively. Four kinetic models were used to analyze the experimental data, and it was determined that the pseudo-second-order kinetic model best described the adsorption process, which involved chemical adsorption and the internal diffusion. Thermodynamic parameters, including the enthalpy, entropy, and Gibbs free energy, were calculated. These parameters indicated that the adsorption of FD on JH was spontaneous and endothermic and the adsorption of FD on SH was unspontaneous and exothermic.

## 1. Introduction

Industrial organic dyes are highly toxic, and therefore, the dyeing industry, which releases 100 tons of dyes in the environment annually, is responsible for the pollution of rivers and springs [1, 2]. Most of the effluents from the textile industries are often loaded with a mixture of dyes, acids, bases, and soluble solids [3]. Once these pollutants are exposed to water, their removal via adsorption becomes difficult owing to their complex molecular structures, which can withstand various environmental conditions. Therefore, more studies on the treatment of aqueous solutions should be conducted to overcome this environmental problem, and methods that could be used to remove even small amounts of different types of synthetic dyes from wastewaters should be considered.

The textile industries often produce liquid wastes that are loaded with mixtures of dyes, acids, bases, and soluble solids [3], making adsorption difficult. Therefore, aqueous solutions treatment is one of the biggest problems we face today. Once the dyes are exposed to water, it is difficult to adsorption them, because they have a complex molecular structure, capable of tolerating various environmental conditions. Moreover, when the concentration of these dyes in water is low, the water is clear. Thus, the decolorization of aqueous solutions that contain different types of synthetic dyes is important.

**Figure 1:**
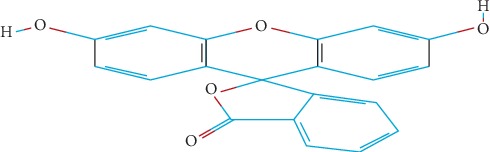
Chemical structure of the fluorescein (C_20_H_12_O_5_) dye.

The chemical structure of resorcinolphthalein or fluorescein dye (FD), which is one of the commercially available textile dyes, is shown in Figure 1.

The molecular formula of FD, which is widely used as artificial coloring agent, is C_20_H_12_O_5_. Fluorescein dye can be obtained by heating resorcinol and phthalic anhydride on a zinc catalyst; the obtained product is a crystalline dark red powder, and its melting point ranges from 314 to 316°C. Resorcinolphthalein is also known as FD owing to its strong green fluorescein alkaline solutions even at low concentration. To date, FD has been used as dye for liquid coloring in analytical devices, in the cosmetics industries, and as aqueous detector.

While no specific method exists for the complete adsorption of dyes from aqueous solutions, some of the traditional methods for removing dyes from aqueous solutions involve biological treatment, coagulation, buoyancy, adsorption, oxidation, and excessive filtration [3], and the most important treatment of all these methods is adsorption.

Adsorption is the process where molecules from aqueous solutions adhere on the surface of the adsorbent surface via physical forces or chemical bonds. Depending on the nature of the adsorbents and the chemical structure of the dyes, adsorption occurs via electrostatic interactions or van der Waals forces.

Many natural materials, including coffee waste, have been used to adsorb various pollutants from aqueous solutions [4, 5]. Large amounts of coffee beans are produced and used worldwide, and coffee waste has been used to adsorb hazardous particles from aquifers and gaseous mixtures or for the desalinization of water [6]. Coffee powder has been used as low-cost additive for the adsorption of a series of rhodamine dyes, such as rhodamine B and rhodamine 6G, from aqueous solutions [7]. *Aleurites moluccanus* seeds have been used for the adsorption of methylene blue dye and rhodamine B from aqueous solutions [8]. Moreover oat hulls [9], date palm fiber [10], and wood apple shells [11] have been used for the adsorption of malachite green from aqueous solutions; water hyacinth [12], date palm fiber [13], and *Calligonum comosum* leaf powder [14] have been used for the adsorption of crystal violet dye from aqueous solution; date palm leaf [15] has been used for the adsorption of Congo red dye from aqueous solution; teak leaf powder [16] has been studied for the adsorption of eosin yellow; and kenaf core fiber has been used for the adsorption of reactive anionic [17] and acidic dyes [18].

The aim of this study was to analyze the kinetics and thermodynamics of the adsorption of FD from aqueous solution using wild herb microparticles.

## 2. Materials and Methods

### 2.1. Materials

We purchased FD from Sigma-Aldrich (St. Louis, MO, USA) and used distilled water to prepare FD solution.

**Figure 2:**
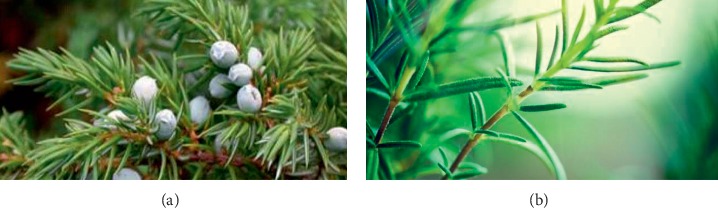
*Juniperus* (JH) and *Solenostemma argel* (Del) *Hayne* (SH).

*Juniperus* (JH; Figure 2), which is an evergreen trees with seedless fruit that typically grows in cold climates, is abundant in southern Saudi Arabia. The branches of JH secrete a dark colored substance that presents medical uses [19].

*Solenostemma argel* (Del) *Hayne* (SH; Figure 2), which is a simple herbaceous plant, blooms in the summer and can be found in many mountainous and desert regions of the Arabian Peninsula and North Africa [20]. Its small white flowers are grouped in tentacles and its fruit is smooth, velvety, fossilized, and dark red.

### 2.2. Preparation of Adsorbents

The JH and SH adsorbents used in this study were purchased from perfumer. The herbs were first dried at 50°C for 30 min, and then they were cut into small pieces and ground to powder using a laboratory planetary ball mill (DECO-PBM-V-0.4L). The powders were sieved into particles less than 50 *μ*m in size using an Octagon D200 digital sieve shaker and were stored in glass bottles for further use without any pretreatment.

### 2.3. Preparation of FD Solution

The FD stock solution (1000 mg/L) was prepared using double distilled water. All solutions used in the experiments were prepared by diluting the stock solution to predetermined concentrations.

### 2.4. Methods

The adsorption experiments were performed in flasks that contained 100 mL FD solution of predetermined concentration and different amounts of adsorbent herbs. The adsorption equilibrium was investigated for different dye concentrations that ranged between 2 and 10 mg/L. In addition, kinetics experiments were conducted using dye with the concentration of 10 mg/L and 0.5 g adsorbent at 25°C. The mixtures were shaken at 120 rpm for 16 h using a Rotaterm orbital and linear shaker.

The initial concentration of dye in this study was 10 mg/L. The effect of the pH on the adsorption of FD was investigated, and the pH was adjusted in the range of 3–12 using either 1 M NaOH or 1 M HCl. A pH meter was used to monitor the changes in pH. In addition, the adsorbent-adsorbate contact time was varied between 30 and 240 min. The effect of the temperature on the adsorption process was studied in the range of 25 to 70°C for 3 h; the amount of adsorbent and the initial dye concentration used for these experiments were 0.5 g and 10 mg/L, respectively, and 10 mg/L, respectively [21]. All mixtures were filtered using 42 micron Whatman filter paper, and the dye concentrations were determined using a UV/Vis spectrophotometer at the wavelength of 450 nm.

The quantity of dye adsorbed at equilibrium (*q*_e_ (mg/g)) was calculated using the following equation [21]:

(1) $$\begin{array}{c} q_{\text{e}}=\frac{\left(C_{0}\;-\;C_{\text{e}}\right)V}{m}, \end{array}$$

where *C*_0_ and *C*_e_ (mg/L) are the initial concentrations of dye and the equilibrium concentration of dye at equilibrium, respectively, *V* (L) is the volume of the solution, and *m* (g) is the mass of adsorbent.

The percentage of FD adsorbed from the solution (Ads_FD_ (%)) was calculated using the following equation [21]:

(2) $$\begin{array}{c} {\text{Ads}}_{\text{FD}}\%=\frac{\left(C_{0}\;-\;C_{\text{e}}\right)}{C_{0}}\times 100. \end{array}$$

Four kinetic models were used to describe the behavior of adsorbent, and the Langmuir and Freundlich isotherm models were employed to study the adsorption data. The following thermodynamic parameters, enthalpy (∆*H*°), entropy (∆*S*°), and free energy (∆*G*°), were calculated, and the results were used to study and interpret the effect of temperature on the adsorption process.

The functional groups that participated in the adsorption processes were determined using Fourier-transform infrared (FTIR) spectroscopy and X-ray diffraction (XRD) analysis. Scanning electron microscopy (SEM) images of the adsorbent surfaces were obtained using a JSM-6380 LA SEM instrument with the high resolution of 3.0 nm.

## 3. Results and Discussion

### 3.1. Characterization of Adsorbents

**Figure 3:**
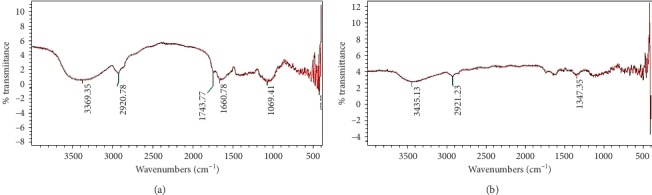
FTIR analysis of the functional groups in (a) JH and (b) SH.

The FTIR spectra of the adsorbents were used to differentiate functional groups, such as –OH (alcohols and carboxylic acids), –CH– (alkanes), –N–H (amines), and C=O (carbonyl), present in the structure of JH and SH (Figure 3), which could form bond with FD during adsorption and therefore could play important roles in adsorption of FD [22, 23]. The absorption peaks identified in the FTIR spectra of JH and SH are summarized in Table 1.

**Figure 4:**
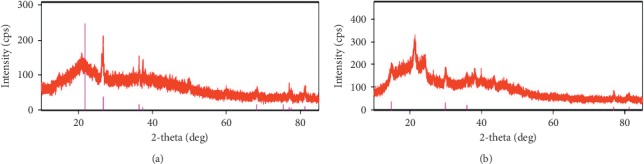
XRD pattern of (a) JH and (b) SH.

A broad hump and a sharp diffraction peak were observed in the XRD patterns of JH and SH (Figure 4), which indicated that the adsorbents consisted of combinations of amorphous and crystalline structures.

**Figure 5:**
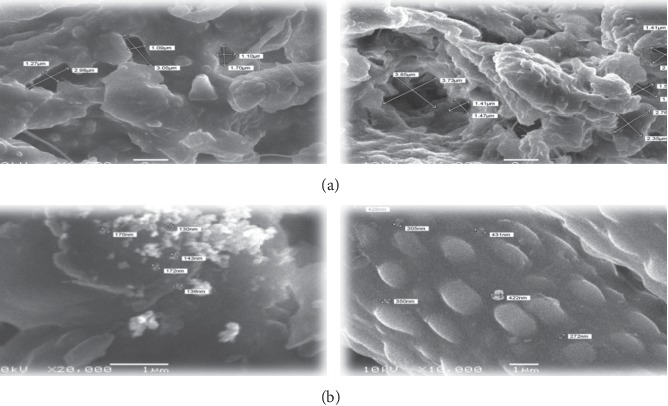
SEM photograph for surface of JH and SH before (a) and after (b) adsorption processes.

The SEM images of the surface of JH and SH adsorbents are presented in Figures 5(a) and 5(b); JH contained deep pores ranging in size from 1.9 to 3.5 *μ*m and the SH contained smaller pores ranging in size from 130 to 350 *μ*m. This explains that the presence of these deep pores with the size of the micrometer greatly aids in adsorption of large quantities of FD molecules on the surface of adsorbents.

### 3.2. Effect of Initial FD Concentration on Adsorption

**Figure 6:**
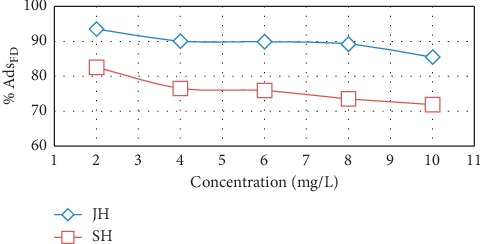
Effect of initial concentration on quantity of FD adsorbed by JH and SH (adsorbent dosage, 0.5 g; solution volume, 100 mL; contact time, 16 h; and temperature, 25°C).

In this study, the initial concentration of FD, which varied from 2 to 10 mg/L, affected the percentage of FD adsorbed. When the initial FD concentration increased, the percentage of adsorbed FD decreased (Figure 6). The lowest percentage of adsorbed FD was associated with the highest initial concentration of FD. At high FD concentrations, the competitive dispersion of FD at the pores available on the adsorbent surface increased [17]; therefore, the pores were closed and the FD molecules were prevented from migrating into the deep pores of the absorbent. Consequently, adsorption occurred only at the surface of the adsorbent [24, 25].

### 3.3. Effect of Adsorbent Dose on Adsorption

**Figure 7:**
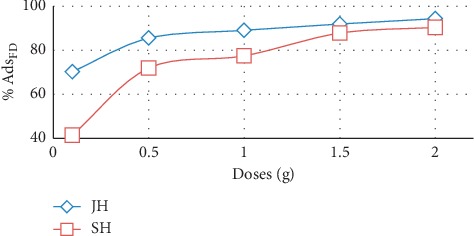
Effect of adsorbent dose on quantity of FD adsorbed by JH and SH (concentration of FD, 10 mg/l; solution volume, 100 mL; and temperature, 25°C).

The effect of the adsorbent dose on the adsorption of FD on JH and SH was studied. The percentage of adsorbed FD increased as the adsorbent doses increased (Figure 7). This was attributed to the increased availability of exchange sites or the increase in the surface area that featured large number of adsorption sites [26].

### 3.4. Effect of Contact Time on Adsorption

**Figure 8:**
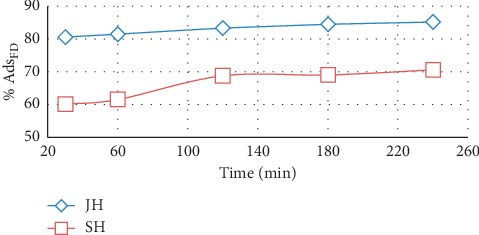
Effect of contact time on quantity of FD adsorbed by JH and SH (adsorbent dosage, 0.5 g; concentration of FD, 10 mg/l; solution volume, 100 mL; and temperature, 25°C).

The adsorption equilibria were analyzed for 30 to 240 min. The FD adsorption rates of both adsorbents (JH and SH) increased in time (Figure 8). The percentage of adsorbed FD increased rapidly during the first 120 min of adsorbent-adsorbate contact, and then the process slowed down for both adsorbents. This occurred because the number of active adsorption sites decreased in time [9, 24].

### 3.5. Effect of pH on Adsorption

**Figure 9:**
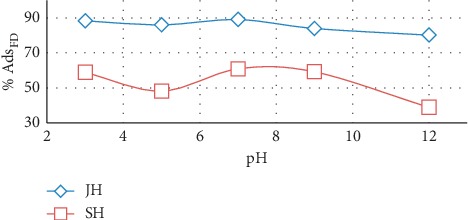
Effect of pH on quantity of FD adsorbed by JH and SH (adsorbent dosage, 0.5 g; concentration of FD, 10 mg/l; solution volume, 100 mL; contact time, 16 h; and temperature, 25°C).

Adsorption experiments were carried out at various pH levels between 3 and 12 to evaluate the effect of the pH of the FD solution on the percentage of FD adsorbed on both JH and SH. The percentage of adsorbed FD decreased slightly as the pH was increased from 3 to 5; as the pH was further increased and exceeded 7, the percentage of adsorbed FD increased for both adsorbents (Figure 9). At pH values exceeding 8, the percentage of adsorbed FD decreased as the pH was further increased.

The pH of the aqueous solution is an important parameter, because it affects the ionization of dye molecules and surface charge of adsorbents [7].

The percentage of adsorbed FD decreased as the pH was increased from 6 to 12. That could be attributed to the adsorption of OH^−^ ions on the surface of the adsorbents causing the surface to become negatively charged. Moreover, the functional groups of the adsorbents, such as the –OH and C=O groups, could occur between these groups and the anionic FD molecules. Consequently, the percentage of adsorbed FD decreased when the pH increased from 6 to 12 [27].

### 3.6. Effect of High Temperature on Adsorption

**Figure 10:**
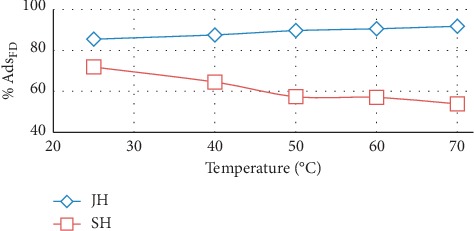
Effect of temperature on quantity of FD adsorbed by JH and SH (adsorbent dosage, 0.5 g; concentration of FD, 10 mg/l; solution volume, 100 mL; and contact time, 4 h.

Our experimental results indicated that when the temperature increased from 25 to 70°C, the percentage of FD adsorbed on JH increased and the percentage of FD adsorbed on SH decreased. All experiments were performed at pH of 8.5 and initial FD concentration of 10 mg/L (Figure 10).

The increase in the percentage of FD adsorbed on JH with the temperature could indicate that FD reacted with the functional groups on the surface of JH [28].

Conversely, the bonds between the FD molecules and functional groups on the surface of SH were physical bonds and could be easily broken at high temperature [29–32].

## 4. Adsorption Isotherm Models

To elucidate the adsorption mechanism of FD from aqueous solutions using JH and SH as adsorbents, we used the Langmuir and Freundlich adsorption isotherm models.

**Figure 11:**
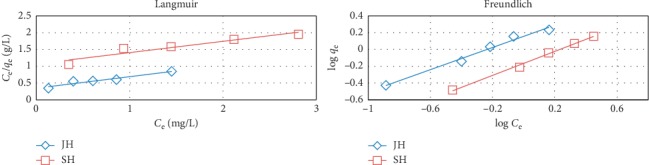
Langmuir and Freundlich isotherms plots for the FD adsorption by JH and SH (adsorbent dosage, 0.5 g; concentration of FD, 10 mg/l; solution volume, 100 mL; contact time, 16 h; and temperature, 25°C).

The Langmuir adsorption isotherm model assumes that adsorption occurs on the surface of the adsorbent and is a monolayer process [33] and could be described as follows [29]:

(3) $$\begin{array}{c} \frac{C_{\text{e}}}{q_{\text{e}}}=\frac{1}{K_{\text{L}}q_{\text{m}}}+\frac{C_{\text{e}}}{q_{\text{m}}}, \end{array}$$

where *C*_e_ (mg/L) is the concentration of adsorbate at equilibrium, *q*_e_ is the adsorption capacity at equilibrium, *q*_m_ is the maximum adsorption capacity of the adsorbate, and *K*_L_ is the adsorption equilibrium constant. The relationships between *C*_e_/*q*_e_ and *C*_e_ for the adsorption of FD on JH and SH were linear (Figure 11), and the slopes of the plots and correlation coefficients (*R*^2^) are listed in Table 2. Experimental results revealed that the Langmuir adsorption isotherm is good with the JH adsorbent and did not fit with the SH adsorbent; moreover, the maximum theoretical adsorption capacities of the adsorbents are greater than the experimental ones [8].

A dimensionless physicochemical constant was applied as separation factor (*R*_L_), which indicated the favorable nature of the adsorption process, and was calculated as follows [30]:

(4) $$\begin{array}{c} R_{\text{L}}=\frac{1}{1+K_{\text{L}}C_{0}}. \end{array}$$

The separation factor *R*_*L*_ for the adsorption of FD on JH ranged from 0 to 1, which indicated the adsorption was favorable (Table 2). Typically, *R*_L_ indicates whether the isotherm type is irreversible (*R*_L_ = 0), favorable (0 < *R*_L_ < 1), linear (*R*_L_ = 1), or unfavorable (*R*_L_ > 1) [30].

The Freundlich adsorption isotherm model is an experimental equation and another form of Langmuir, which can be applied to the multilayer adsorption process [33] and could be described as follows [30]:

(5) $$\begin{array}{c} \log\;q_{\text{e}}=\log\;K_{\text{F}}+\frac{1}{n}\log\;C_{\text{e}}, \end{array}$$

where *n* is the Freundlich equilibrium coefficient. The plot of the relationship between log *q*_e_ and log *C*_e_ is presented in Figure 11, and the slope and *R*^2^ values are listed in Table 2. The *R*^2^ values indicated that the Freundlich model fitted the experimental results better than the Langmuir one. The magnitude *n* can be used to assess the suitability of the adsorption process. When *n* ranged from 1 to 2, the adsorption of FD on JH and SH was somewhat difficult [30].

The obtained results revealed that the adsorption of FD on the surface of JH and SH was a multilayer process.

## 5. Adsorption Kinetic Models

The pseudo-first-order, pseudo-second-order, intraparticle diffusion, and liquid film diffusion kinetic models were used to elucidate the adsorption mechanism of FD on JH and SH and to determine the rate dominant step of the adsorption process.

The pseudo-first-order model (PFO) could be described as follows [28]:

(6) $$\begin{array}{c} \log\left(q_{\text{e}}-q_{t}\right)=\log\;q_{\text{e}}-\frac{K_{1}t}{2.303}, \end{array}$$

where *q*_e_ and *q*_*t*_ represent the quantity of FD adsorbed at equilibrium and at the time, *t*, respectively, and *K*_1_ is the rate constant of the pseudo-first-order reaction.

The pseudo-second-order model (PSO) could described as follows [28]:

(7) $$\begin{array}{c} \frac{t}{q_{t}}=\frac{1}{K_{2}q_{\text{e}}^{2}}\;+\frac{t}{q_{\text{e}}}, \end{array}$$

where *K*_2_ is rate constant of the pseudo-second-order reaction.

The intraparticle diffusion (IPD) coefficient *K*_ipd_ could be calculated using the following equation [28]:

(8) $$\begin{array}{c} \;q_{t}=K_{\text{ipd}}\;t^{1/2}-C, \end{array}$$

where *C* is a constant that provides information on the thickness of the boundary layer.

The liquid film diffusion model (LFD) could described as follows [28]:

(9) $$\begin{array}{c} -\ln\left(1-F\right)=K_{\text{lfd}}t-C, \end{array}$$

where *F* = *q*_t_/*q*_e_, *K*_lfd_ is the rate constant of the liquid film diffusion reaction, *t* is the time, and *C* is a constant related to the boundary layer.

**Figure 12:**
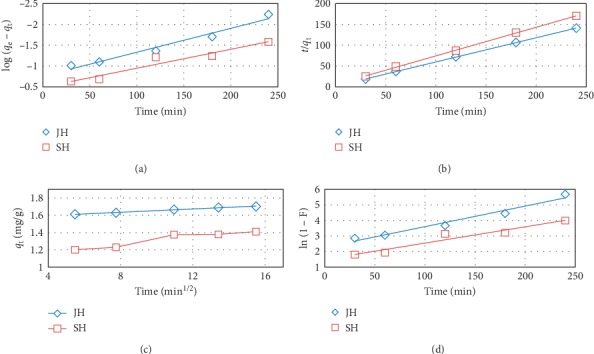
Plot of adsorption kinetic model ((a) PFO, (b) PSO, (c) IPD, and (d) LFD) for the FD adsorption by JH and SH (adsorbent dosage, 0.5 g; concentration of FD, 10 mg/l; solution volume, 100 mL; and temperature, 25°C).

The linear plots of the four kinetic models for the adsorption of FD on JH and SH are presented in Figure 12. The parameters, constants, and *R*^2^ values for the four kinetic models are listed in Table 3. The experimental *q*_e_ values of FD adsorbed did not agree with the calculated ones, and therefore the pseudo-first-order model did not fit well for the experimental data [12, 22]. However, the pseudo-second-order model fits well the kinetic data [28]. Furthermore, the results also revealed that the adsorption process involved the exchanging or sharing of electrons between FD and JH and SH, and this was the rate dominant step of the adsorption [27]. Further, the parameters of the intraparticle diffusion and the liquid film diffusion model, indicating that they are some of the factors determining the rate in the FD adsorption process (Table 3). The process consists of two steps or more, the first step being full diffusion on the adsorbent surface, the second step being the gradual diffusion of the particles into the pores, and the third step, which is the final equilibrium step. Figure 12 shows that the linear plots of dye adsorption did not pass through the original, indicating that FD adsorbed on the JH and SH was controlled by intraparticle diffusion and more than one mechanism is involved in adsorption. These results also indicated that JH was a better adsorbent for FD than SH.

## 6. Thermodynamics of Adsorption

**Figure 13:**
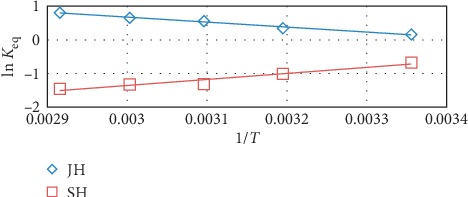
Van't Hoff plots for the FD adsorption by JH and SH (adsorbent dosage, 0.5 g; concentration of FD, 10 mg/l; solution volume, 100 mL; and contact time, 4 h.

The thermodynamic parameters, ∆*S*°, ∆*H*°, and ∆*G*°, were calculated using the following equations [34]:

(10) $$\begin{array}{c} K_{\text{eq}}\;=\;\frac{q_{\text{e}}}{C_{\text{e}}},\\ \ln\;K_{\text{eq}}\;=\;\frac{\Delta S^{{}^{\circ}}}{R}-\;\frac{\Delta H^{{}^{\circ}}}{RT},\\ \Delta G^{{}^{\circ}}=\Delta H^{{}^{\circ}}-T\Delta S^{{}^{\circ}}, \end{array}$$

where *K*_eq_ is the equilibrium constant, *q*_e_ (mg/g) is the quantity of FD adsorbed at equilibrium, *C*_e_ (mg/L) is the concentration of FD in solution at equilibrium, *R* is the universal gas constant (8.314 mol/K), and *T* (K) is the absolute temperature of the solution. The Van't Hoff plots of ln *K*_eq_ vs. 1/*T* are illustrated in Figure 13, and ∆*S*°, ∆*H*°, and ∆*G*° values are summarized in Table 4. The ∆*H*° and ∆*S*° values were determined from the slopes and intercepts of the plots. The positive ∆*H*° and ∆*S*° values for the adsorption of FD on JH demonstrated the endothermic nature of the adsorption process. Conversely, the ∆*H*° and ∆*S*° values for the adsorption of FD on SH indicated that the process was exothermic and unspontaneous of the adsorption process [27, 34].

The negative *ΔG*° values for the adsorption of FD on JH indicated that the adsorption was feasible and spontaneous. In addition, ∆*G*° ranged from—20 to 0 kJ/mol which indicated that the predominating adsorption mechanism was physical in nature [30, 35]. The positive ∆*G*° value for the adsorption of FD on SH indicated that the adsorption was unspontaneous.

## 7. Conclusion

In this study, the analysis of JH and SH, which were used as adsorbents, revealed the presence of –OH (alcohols and carboxylic acids), –CH– (alkanes), –N–H (amines), and C=O (carbonyl) groups in their structures, and these groups participated in the adsorption of FD. The XRD patterns of JH and SH revealed that their structures were combinations of amorphous and crystalline phases. The SEM images of the surfaces of JH and SH revealed the presence of many deep micropores, which could adsorb large quantities of FD. The Langmuir and Freundlich isotherm models were used to describe the adsorption of FD on JH and SH. The equilibrium data fitted well the Freundlich model. The percentage of FD adsorbed from aqueous solutions on JH was 85.5%, which was higher than the percentage adsorbed on SH (71.9%). The *n* and *R*_L_ values ranged from 1 to 2 and from 0 to 1, respectively; moreover, *q*_m_ was determined to be 2.91 and 2.565 mg/g for JH and SH, respectively. The kinetics of the adsorption FD on JH and SH were examined using the pseudo-first-order, pseudo-second-order, intraparticle diffusion, and liquid film diffusion models and was concluded that the adsorption followed the pseudo-second-order kinetic model, which involved chemical adsorption and the internal diffusion. The positive ∆*H*° and ∆*S*° values and negative Δ*G*° value associated with the adsorption of FD on JH confirmed that the process was endothermic and spontaneous. Conversely, the negative ∆*H*° and ∆*S*° values and positive Δ*G*° value associated with the adsorption of FD on SH suggested that the process was exothermic and unspontaneous. These results also indicated that JH was better than adsorbent for FD than SH. Further, the adsorption of dyes from aqueous solutions requires more studies.

## Figures and Tables

**Table 1:** FT-IR peaks of the functional groups in JH and SH.

Adsorbent	Peaks (cm^−1^)	Functional groups
JH	3392.81–3423.84, 2913.05–2925.31, 1625.25–1728.04, 1078.93–1016.49	–OH, –COOH, –N–H, –C–H, C=O
SH		

**Table 2:** Adsorption isotherm parameters.

Adsorption isotherm models	Parameter	Adsorbent	
		JH	SH
Langmuir model	*q*_m_ (mg/g)	2.912	2.565
	*K*_L_ (L/mg)	0.993	0.376
	*R*_L_	0.091	0.210
	*R* ^2^	0.936	0.867

Freundlich model	*K*_F_ (mg^(1−1/*n*)^ g^−1^ L^1/*n*^)	1.433	0.669
	*n*	1.514	1.470
	*R* ^2^	0.985	0.987

**Table 3:** Parameters of adsorption kinetic models.

Kinetic models	Adsorbent	
	JH	SH
*q*_e,exp_, mg g^−1^	1.710	1.438

Pseudo-first-order		
*q*_e,cal_, mg g^−1^	0.174	0.273
*k*_1_, g/mg min	0.013	0.008
*R*^2^	0.969	0.853

Pseudo-second-order		
*q*_e,cal_, mg g^−1^	1.720	1.436
*k*_2_, g/mg·min	0.197	0.097
*h*, mg g^−1^·min	0.583	0.200
*t*^1/2^	16.56	9.28
*R*^2^	0.999	0.999

Intraparticle diffusion		
Step 1		
*k*_ipd_, mg g^−1^·min^1/2^	0.010	0.033
*C*, mg g^−1^	1.555	1.006
*R*^2^	0.990	0.927

Step 2		
*k*_ipd_, mg g^−1^·min^1/2^	0.008	0.003
*C*, mg g^−1^	1.576	1.338
*R*^2^	0.991	0.907

Liquid-film diffusion		
*K*_lfd_, l/min	0.013	0.008
*C*, mg g^−1^	2.283	1.661
*R*^2^	0.969	0.853

**Table 4:** Thermodynamic parameters.

Parameter	Adsorbent	
	JH	SH
∆*H*° (kJ/mol)	12.27	−14.87
∆*S*° (J/mol K)	42.41	−55.89
∆*G*° (kJ/mol) at 298 K	−0.37	15.17
*R* ^2^	0.990	0.944

## Data Availability

The data used to support the findings of this study are included within the article.
